# Physical Changes of Biomacromolecules upon Covalent Surface Immobilization

**DOI:** 10.1021/acs.langmuir.5c06836

**Published:** 2026-03-12

**Authors:** Bianca Mercado Velez, Vaishali Sharma, Seth Kriz, Erico T. F. Freitas, Paul Goetsch, Caryn L. Heldt

**Affiliations:** † Department of Biological Sciences, 3968 Michigan Technological University, Houghton, Michigan 49931, United States; ‡ Health Research Institute, Michigan Technological University, Houghton, Michigan 49931, United States; § Department of Chemical Engineering, Michigan Technological University, Houghton, Michigan 49931, United States; ∥ Materials Characterization and Fabrication Facility, Michigan Technological University, Houghton, Michigan 49931, United States

## Abstract

Immobilization of large biomacromolecules is often required for analytical quantification and physicochemical characterization. However, immobilization can alter the structure and size of the particles being studied. Here, two exosomes (derived from HEK-293 and MDA-MB-231 cells) and three viral particles (Suid herpesvirus 1 (SuHV), xenotropic murine leukemia virus (XmuLV), and porcine parvovirus (PPV)) were immobilized to different covalent chemistries to understand how surface chemistry influences particle deformation during immobilization. The surface chemistries explored were: (i) NHS (*N*-hydroxysulfosuccinimide) and EDC (1-ethyl-3-(3-(dimethylamino)­propyl) carbodiimide hydrochloride), and (ii) poly l-lysine (PLL) and glutaraldehyde (GA). Morphological changes in biomolecules following immobilization were quantified by measuring the height-to-diameter (*H*/*D*) ratios attained from atomic force microscopy (AFM) topographic images. These observations were further supported by complementary size and morphology analyses using dynamic light scattering (DLS) and liquid phase transmission electron microscopy (TEM). NHS/EDC chemistry consistently resulted in more significant particle flattening than PLL/GA, as evidenced by lower average *H*/*D* ratios across all biomacromolecules. Greater flattening effects were observed on the soft lipid envelope of exosomes as compared to viruses, due to differences in structural rigidity. Both immobilization chemistries resulted in a lower *H*/*D* ratio in tumor-derived MDA-MB-231 exosomes compared to nontumor-derived HEK-293 exosomes, likely due to the known softer mechanical properties of tumor-derived exosomes. Furthermore, immobilization of the enveloped viruses SuHV and XMuLV with NHS/EDC exhibited flattening effects and lower *H*/*D* ratios. Immobilization of nonenveloped PPV resulted in a low *H*/*D* ratio on NHS/EDC, which was likely due to particle aggregation rather than deformation. These findings provide valuable guidance for selecting appropriate surface chemistries for nanoscale biointerface studies and offer implications for surface-based diagnostics, high-throughput biosensing, and nanomaterial functionalization.

## Graphical abstract

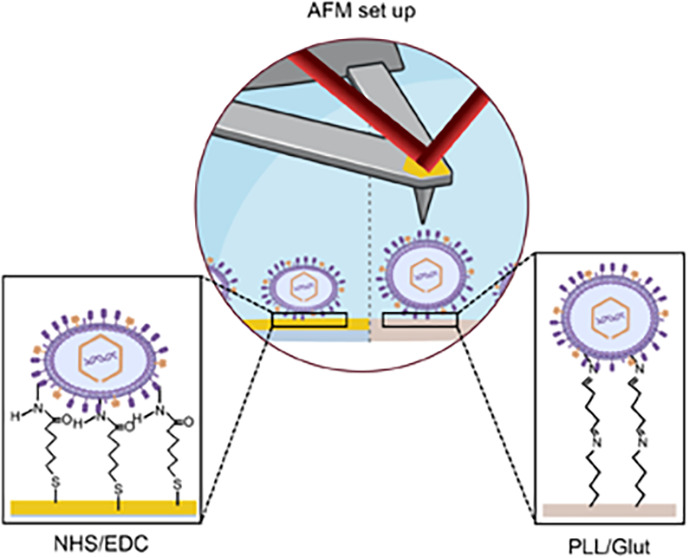

## Introduction

Physicochemical and biological characterization of biomacromolecules, like exosomes and viruses, is crucial to ensure they are safe, effective, and reliable for clinical use, whether as drug delivery vehicles or diagnostic markers. Bulk characterization techniques provide information on the size, concentration, and surface properties of these biomacromolecules.^1^ However, many of these techniques provide population-level data that overlooks heterogeneity within the sample.^1,2^ In contrast, single-particle analysis offers high-resolution structural and functional insights, capturing population heterogeneity.^3,4^ Some single-particle analyses, like conventional transmission electron microscopy (TEM) requires vacuum conditions, restricting the ability of researchers to characterize biomacromolecules in their native physiological state.^5,6^ In contrast, atomic force microscopy (AFM) enables single-particle analysis in liquid environments, providing real-time imaging and measurements of molecular interactions under conditions that closely mimic the biomacromolecule’s native physiological state.

AFM is a versatile method for investigating the physicochemical properties and interactions of biomolecules at the nanoscale, but a critical aspect of the method is the effective immobilization of biomolecules onto a substrate. Electrostatic interactions are simple immobilization methods and are suited for imaging and elasticity measurements by nanoindentation.^7–9^ However, to measure molecular interactions by force spectroscopy, a strong attachment of the biomolecule to the substrate with covalent immobilization is required.^9,10^ Force spectroscopy has proven valuable in understanding protein–ligand binding.,^11–14^ DNA–protein interactions,^15,16^ and cell adhesion.^17–20^ This technique is increasingly applied to complex biomacromolecules, such as viruses and exosomes, to characterize their physicochemical properties and assess the heterogeneity within populations.^21–25^

Various covalent immobilization strategies are available to stably attach biomolecules to solid substrates for biophysical and surface interaction studies. These chemistries enable durable and site-specific linkages between the functionalized surface and immobilized biomolecules. One common method is silanization of glass and silicon surfaces, using agents like APTES ((3-aminopropyl)­triethoxysilane) to coat a surface with primary amine (NH_2_) groups, which serve as covalent linking points.^26,27^ Another distinct and versatile strategy, often used for gold surfaces, is the formation of a self-assembled monolayer (SAM)^28^ with a multifunctional carbon chain. A thiol molecule on one end will bond to the gold, the carbon chain will self-assemble into a monolayer (SAM), and the free end can be functionalized with amines, carboxylic acids, and other groups, providing versatility. When a carboxylic acid is the end group on SAM, carbodiimide-mediated coupling (EDC/NHS) attach lysine on the biomolecule to the surface of the SAM.^25,29^ Additionally, cross-linker reagents, like glutaraldehyde (GA)^30,31^ are frequently employed, often in combination with other surface functionalization steps, to achieve controlled covalent immobilization.^32–34^ These covalent chemistries allow for the stable attachment of varied biomolecules, including nonenveloped viruses and protein capsids, for biophysical and force interaction studies.^35,36^ However, different covalent immobilization methods may induce structural changes, particularly when immobilizing biomacromolecules with lipid bilayer membranes. Any physical change could influence the force measurements and potentially convolute the interpretation of the results.^34,37^

Covalent immobilization of lipid membranes for AFM studies affects the native structure and fluidity of lipid bilayers. The effects on the lipid bilayers can occur due to the formation of chemical bonds that anchor membrane components directly to the substrate.^34^ These disruptions may alter the membrane curvature, lateral mobility, or integrity, potentially affecting the biological function and/or the biophysical properties of the biomolecules.^38–41^ Such effects become especially significant when immobilizing complex lipid-based nanostructures like exosomes and viruses. Exosomes immobilized with APTES-functionalized mica demonstrated membrane deformity and rupture due to the silanization of the substrate.^42^ Although covalent immobilization has been utilized for force studies on SARS-CoV-2 through attachment of angiotensin-converting enzyme 2 (ACE2) to a gold surface with NHS/EDC chemistry, little is known about the actual effects of the attachment methods on the structure of the virus and exosomes.^43,44^ Therefore, it is essential to evaluate how the currently employed covalent immobilization chemistries may impact the structural integrity and native conformation of lipid-containing biomolecules.

We are interested in understanding the physicochemical properties of large biomacromolecules (exosomes and viruses) for application in biotherapeutics. These complex biologics can encapsulate proteins, nucleic acids, and possess heterogeneous molecular membranes, exhibiting diverse compositions and curvatures. Exosomes are lipid bilayer vesicles that carry cargo specific to the parent cell type, including cancerous cells, and vary in size, ranging from 30 to 150 nm in diameter.^45,46^ Similarly, viruses range from 20 nm to several 100 nm in diameter. Enveloped viruses have a lipid membrane surrounding a protein capsid encapsulating genetic material. In contrast, nonenveloped viruses are more simple and are comprised of only a protein capsid and genetic material.^47^ They are often heterogeneous in size and cargo. However, further understanding of the physicochemical properties of these biomacromolecules is needed to improve current isolation and detection methods, such as chromatography, precipitation, and biosensor design.

Using AFM, we determined the structural effects that follow two covalent immobilization chemistries, gold functionalized NHS (*N*-hydroxysulfosuccinimide) and EDC (1-ethyl-3-(3-(dimethylamino)­propyl) carbodiimide hydrochloride) chemistry, was compared to mica functionalized with poly l-lysine (PLL) and glutaraldehyde (GA). We assessed the immobilization efficacy of chemistries with exosomes derived from noncancerous (HEK-293 cells) and cancerous (MDA-MB-231 cells) cells, as well as different enveloped and nonenveloped viruses. Cancer-derived exosomes are more prone to deformation due to differences in mechanical properties.^42,48,49^ The viruses were enveloped Suid herpesvirus 1 (SuHV) and xenotropic murine leukemia virus (XMuLV), and nonenveloped porcine parvovirus (PPV).^50–52^ This study explored the structural heterogeneity of the varied biomolecules and deformation differences upon immobilization. Additionally, the findings of this study aid in understanding the effects of covalent immobilization chemistry on the structure of biomolecules for future application of covalent attachment for CFM studies to characterize the physicochemical properties of biomolecules and improve diagnostics and drug delivery.

## Materials & Methods

### Biomolecules

Purified exosomes of 1 × 10^6^ particles per vial (50 μg protein) from human embryonic kidney cells (HEK-293) and triple-negative breast cancer MDA-MB-231 cell lines were purchased from System Bioscience (Palo Alto, CA) and used as received.

PPV (Porcine parvovirus) was propagated in PK-13 (porcine kidney cells, cat# CRL-6489, ATCC American Type Culture Collection, Manassas, VA) in Eagle’s minimum essential media (EMEM) (Invitrogen, Waltham, MA) as described previously.^53^ The viral stock was clarified from cell debris by centrifugation in an ST16R centrifuge (Thermo Fisher Scientific) using a TX-400 swing bucket rotor at 4752*g* at 4 °C for 15 min. The PPV stock was spin-filtered through a 10 kDa Amicon Ultra-4 centrifugal filter to exchange the buffer to phosphate-buffered saline (PBS, Gibco, Grand Island, NY). The buffer-exchanged PPV stock was stored at −80 °C until use.

SuHV-1 (Suid herpesvirus 1 strain Aujeszky, ATCC VR-135) was propagated in Vero cells (ATCC, CCL-81), as described previously.^54^ After 5 days of incubation, the viral stock was collected after two freeze–thaw cycles at −20 °C and room temperature, and clarified by centrifugation at 5500*g* for 15 min at 4 °C. SuHV stock was buffer-exchanged into PBS, like PPV, and stored with 10% glycerol (≥99%) (Sigma-Aldrich, St. Louis, MO) at −80 °C until use.

M.dunni cells persistently infected with XMuLV (Xenotropic murine leukemia virus) were purchased from ATCC (VR-1447). The cells were grown in EMEM supplemented with 10% FBS and 1% pen/strep at 37 °C with 5% CO2 and 100% humidity. After 3–4 days of incubation, the supernatant was collected and centrifuged at 5500*g* for 15 min at 4 °C. The viral stock was collected and concentrated overnight at 4 °C using the Retro X concentrator (Takara, Kusatsu, Japan, cat. #631456). The virus was recovered in the pellet after centrifugation at 1500*g* for 45 min at 4 °C. The pellet was dissolved in PBS, and the virus stock was stored with 10% glycerol at −80 °C until further use.

### Control Surfaces and Covalent Immobilization of Biomolecules

We assessed the efficiency of two covalent immobilization chemistries to immobilize viruses and exosomes. The first chemistry tested was NHS/EDC. Glass slides were sputter-coated with chromium (5 nm) and gold (35 nm) layers to obtain gold-coated slides using a Randex PerkinElmer sputtering system (PerkinElmer, Waltham, MA). For the control surface, gold-coated slides were incubated overnight in ethanol, thoroughly rinsed with nanopure water, and dried in a fume hood. For immobilization, clean and dry gold-coated slides were incubated in a 2 mM or 4 mM solution of 12-mercapto dodecanoic acid (HS­(CH_2_)_11_COOH) and 1-dodecanethiol (HS­(CH_2_)_11_CH_3_) (SigmaAldrich, St. Louis, MO) at a 1:1 ratio in ethanol^25,29^ to create a self-assembled monolayer (SAM). For biomolecule immobilization, the gold-coated slides were functionalized with a SAM at either a 2 mM or 4 mM SAM concentration. After overnight incubation, the SAM functionalized gold slides were equilibrated in nanopure water for 15 min. A solution of 0.1 M NHS (sulfo-NHS (*N*-hydroxysulfosuccinimide) and 0.4 M EDC (1-ethyl-3-(3-(dimethylamino)­propyl) carbodiimide hydrochloride)) from Thermo Fisher Scientific (Waltham, MA) in nanopure water at a volume of 0.5 mL was added to the surface and incubated for 30 min.^25,29^ After the slide was thoroughly rinsed with 1× PBS, pH 7.2, a 15 μL exosome or a 40 μL virus sample was added and incubated for 30 min, followed by rinsing with 1× PBS, pH 7.2. All samples were incubated in 1× PBS, pH 7.2, until AFM imaging, which was performed within 24 h of sample preparation.

The second immobilization chemistry tested was cross-linking poly l-lysine (PLL) with glutaraldehyde (GA). For control surfaces, 12 mm muscovite mica discs (V-1 quality, Sigma-Aldrich, St. Louis, MO) were fixed to a glass slide with either super glue or epoxy. The mica was freshly cleaved by peeling the top few layers with Scotch tape. (3M, St. Paul, MN). Control surfaces of freshly cleaved mica were prepared at the same time. Then, 100–200 μL of 0.01% (w/v) PLL (300 grade, cat# P4707, Sigma-Aldrich, St. Louis, MO) was added to the newly cleaved surface for 30 min. Mica functionalized with PLL as a control surface was prepared following the same protocol. Following incubation, 100–200 μL of 0.2% GA (Sigma-Aldrich, St. Louis, MO) in nanopure water was incubated for 30 min. For biomolecule immobilization, after the mica surface was functionalized with PLL and GA, like the control surfaces, for sample incubation, either 15 μL of exosome or 50–100 μL of virus sample was added to the surface and incubated for 30 min. Between each incubation step, the surfaces were thoroughly rinsed with 1X PBS pH 7.2 (Thermo Fisher Scientific, Waltham, MA).^30,31^

### Contact Angle Measurements

The contact angle of the control surfaces was measured using the sessile drop method. Samples of bare gold, bare mica, gold functionalized with 2 mM and 4 mM SAM, and mica functionalized with PLL, were prepared in triplicate following the same protocol as preparation of control surfaces.^25,29,55^ A 10 μL droplet of nanopure water was placed onto the surface with a microsyringe. Images were obtained with a Ramehart Goniometer model 250 (Ramehart Instrument Co., Succasunna, NJ), and the data were analyzed with the DROP image Advanced software (Succasunna, NJ). Contact angle measurements were performed in triplicate.

### Topographic and Height Analysis with Atomic Force Microscopy (AFM)

Topographic images of exosomes and viruses were obtained using the Asylum Research MFP-3D Origin+ AFM (Oxford Instruments, Santa Barbara, CA) in AC tapping mode and under liquid conditions in PBS pH 7.2. AC tapping mode was selected to minimize biomolecule deformation.^56,57^ Silicon nitride DNP-S10 probes (radius, 10 nm; front and back angles of 15 and 25°, respectively, frequency, 23 kHz; and spring constant, 0.12 N/m) from Bruker (Billerica, Massachusetts) were used to perform 1 × 1 μm scans of samples at scan rates of 0.5–1 Hz with 256 × 256 pixels. Topographic and height analysis scans of the control surface were done across three different scan areas. The average and standard deviation of the surface roughness for the 1 × 1 μm scans of the control surfaces were calculated from root-mean-square (RMS) values in Igor Pro (WaveMetrics, Portland, OR) and AR SPM Software (Santa Barbara, CA).

Particle analysis was done with Igor Pro. AFM topographic images were processed by iterative masking of the regions of interest, explicitly targeting virus particles or exosomes. A first-order flattening and line removal were applied to correct for background tilt and curvature. Particle height was measured using the “Analyze” function in the AR SPM software. A vertical line was drawn across the center for each selected particle to generate a height profile, from which the maximum height was identified.

Lateral dimensions obtained from AFM images were corrected using the Garcia geometrical model to account for lateral broadening introduced by the AFM tip radius. Equations 1 and 2 were applied to the height profile generated from the AFM height analysis to correct the lateral broadening effects of the AFM tip (Figure S1A). Depending on the height of the particle, eq 1 (Figure S1B) was applied for (*H* > *R_t_*), where *R*_t_ is the radius of the AFM tip, and this condition was valid for MDA-MB-231, SuHV, and XMuLV^58,59^ and eq 2 (*H* ≅ *R_t_*) was used for PPV and HEK-293 to calculate the radius of the particle. The corrected diameter for all the particles was calculated using eq 5. Viruses and exosomes are spherical in solution; however, when immobilized to a substrate, the shape of the biomacromolecules is ellipsoid or semispherical.^49,60,61^ Although the model assumes an idealized spherical geometry, it was applied here as a first-order correction to minimize systematic overestimation of lateral dimensions due to the tip radius and geometry. The correction was not intended to reconstruct exact particle geometry, particularly for particles exhibiting ellipsoidal or partially deformed morphologies.

1 $$R_{p}=\left(\frac{FWHM-C_{1}H}{C_{2}}\right)-R_{t}$$

2 $$R_{p}=\frac{\left(\mathrm{FWHM}\right)2-4\left(H\right)2}{8H}-R_{t}$$

3 $$C1=\frac{1}{\tan\left(90-\beta \right)}+\frac{1}{\tan\left(90-\alpha \right)}$$

4 $$C2=\frac{\sqrt{{1+\tan\left(90-\beta \right)}^{2}}-1}{\tan\left(90-\beta \right)}+\frac{\sqrt{{1+\tan\left(90-\alpha \right)}^{2}}-1}{\tan\left(90-\alpha \right)}$$

5 $$D_{p1}=2R_{p}$$

*R*_*p*_ is the radius of the immobilized particle, FWHM is the full width at half-maximum height, *H* is the maximum height of particle, *C1* and *C2* are coefficients determined using the front (α) and back (β) angles of the AFM tip in eqs 3 and 4, and *D*_*p1*_ is the diameter of the particle immobilized to substrate.

To determine the particle diameter in solution, the height and diameter of the immobilized particles were used to calculate the particle volume, as shown in eq 6. Particle volume was then used to estimate the radius of the particle as if it were spherical in solution, shown in eq 7. Further, the diameter of the particle in solution (*D*_*p2*_ *)* was calculated as the diameter of the immobilized particle using eq 8,

6 $${\mathrm{volume}}_{\mathrm{elipsoid}}=\frac{4}{3}\pi {\left(R_{p}\right)}^{2}c$$

where *c* is 1/2 of the maximum height of the immobilized particle.

7 $$R_{s}=\sqrt[{3}]{\frac{3}{4}{\mathrm{volume}}_{\mathrm{elipsoid}}\pi }$$

8 $$D_{p2}=2R_{p}$$

### Morphological Characterization and Diameter of Biomolecules with DLS and TEM

The diameter of exosomes and viruses in solution was determined by dynamic light scattering (DLS) with a Malvern Zetasizer Nano ZS (Worcestershire, UK) using a 633 nm red laser and low-volume 40 μL cuvettes from Malvern Panalytical. Samples and controls were prepared in triplicates to determine the average and standard deviation of the hydrodynamic diameter. Data was analyzed using the Zetasizer Software version 8.02.

For liquid phase TEM imaging, 1 μL of exosome sample or virus sample were added to a 0.25*0.25 mm well of 50 nm PELCO Silicon Nitride Support Films (PELCO NetMesh) and then covered with a PELCO Ultrathin (3 nm thick) Carbon Film Supported by Lacey Carbon Film on a 400 mesh Copper Grid. Both SiN and carbon support films were plasma treated in the presence of 75% argon and 25% oxygen with a Fischione Plasma Cleaner M1400 (PA) at a low frequency for 15 s before loading the sample. The TEM micrographs were generated with an FEI 200 kV Titan Themis scanning transmission electron microscope (S-TEM) operated at 200 kV and 10^–5^ mBar.

### Statistical Analysis

Statistical analysis was performed to compare the height and diameter of biomolecules immobilized by NHS/EDC and PLL/GA chemistries. Individual particle measurements obtained from AFM images were treated as independent observations. Because of unequal sample sizes and non-normal data distribution, statistical significance was evaluated using a two-tailed Mann–Whitney *U* test (*t*-Test: Two-Sample Assuming Unequal Variances) in Microsoft Excel. Differences were considered significant at *p* < 0.05.

## Results and Discussion

### Model Biomolecules and Covalent Immobilization Methods

We were interested in understanding the effect of two covalent immobilization chemistries on the structure of exosomes and viruses after immobilization. We tested nontumor-derived HEK-293 ^62–64^ and tumor-derived MDA-MB-231 exosomes from a highly metastatic breast cancer cell line,^49^ illustrated in Figure 1A. Both exosome populations exhibit an estimated diameter of 30–150 nm. The lipid bilayers of the exosomes incorporate surface proteins, which mediate interfacial interactions, and also encapsulate molecular cargo such as nucleic acids, proteins, and lipids within their lumen.^65^ Previous AFM studies evaluating exosome mechanics have demonstrated that exosomes originating from tumor cells exhibit greater elasticity and reduced stiffness, reflecting the physiological characteristics of their parent cells.^42^ The viruses used in these studies were the enveloped Suid herpesvirus (SuHV, 110–200 nm), enveloped xenotropic murine leukemia virus (XMuLV, 80–110 nm), and nonenveloped porcine parvovirus (PPV, 20–25 nm),^66–68^ as shown in Figure 1B. Enveloped viruses consist of a lipid bilayer embedded with surface and envelope proteins that enclose a protein capsid encapsulating the viral genome. In contrast, nonenveloped viruses lack a lipid bilayer, which makes enveloped particles more susceptible to structural deformation upon immobilization.^69^ Understanding how covalent immobilization chemistries affect biological particles is essential for accurately characterizing their structural and mechanical properties.

**Figure 1:**
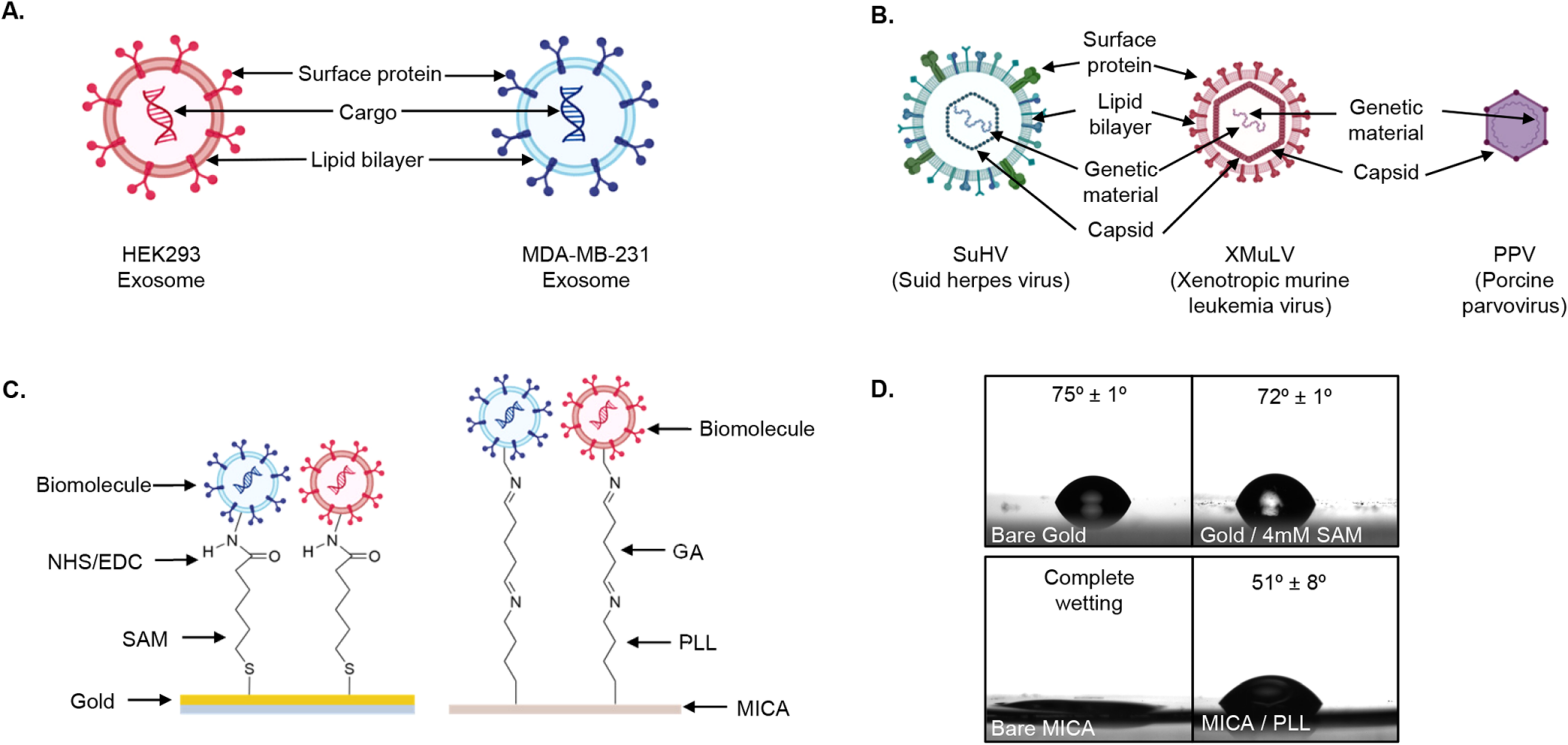
(A) Structure of model HEK293 and MDA-MB-231 exosomes. (B) Structure of model viruses (left to right): SuHV, XMuLV, PPV. (C) Schematic of biomolecule immobilization methods used for AFM studies: gold functionalized with a self-assembled monolayer (SAM) and NHS/EDC chemistry, and mica functionalized with poly l-lysine and glutaraldehyde (PLL/GA). (D) The contact angle of control surfaces: bare gold, gold functionalized with 4 mM SAM, bare mica, and mica functionalized with PLL. Images A–C created with Biorender and ChemDraw.

Biomolecules were first immobilized with NHS/EDC chemistry. Gold substrates were functionalized with SAM. The thiol group on one end of SAM formed a covalent bond with gold, while the other end contained either a methyl or a carboxylic acid group.^25^ The carboxylic acids were functionalized with NHS/EDC, and the NHS esters then created a covalent linkage with the primary amine groups on the biomolecule surface proteins, as shown in Figure 1C. A ratio of 1:1 methyl to carboxylic acid capped SAM linker was utilized to control the density of attached biomolecules on the surface. Previous studies used the NHS/EDC chemistry to immobilize both enveloped and nonenveloped viruses.^25^ However, enveloped-virus immobilization via NHS/EDC chemistry was low (unpublished data); therefore, an alternative immobilization method was sought.

For the second chemistry, mica was functionalized with PLL/GA. PLL tightly associates with mica through electrostatic interactions.^70,71^ After coating the mica with PLL, glutaraldehyde (GA), a bifunctional linker with aldehyde groups at both ends, covalently attached biomolecules to PLL by reacting with amines on PLL and lysine residues on the biomolecules,^72^ as shown in Figure 1C.

Contact angle measurements of the control surfaces characterized the hydrophobic properties of the functionalized substrates. Both bare and mica functionalized with PLL were more hydrophilic than the gold surfaces (Figure 1D). The hydrophilic properties of mica are expected, as prior studies of contact angle on mica had a contact angle of less than 10°, signifying mica is a hydrophilic surface.^73^ The mica functionalized with PLL had a larger contact angle than bare mica, resulting in a decrease in the hydrophilic properties of the surface. However, functionalization of gold with 4 mM SAM reduced the contact angle relative to bare gold, reflecting increased hydrophilicity caused by polar functionalities introduced by the self-assembled monolayer. The control surfaces from the NHS/EDC chemistry were more hydrophobic than those of PLL/GA. The observed changes in contact angle confirm successful surface functionalization and reflect differences in surface wettability between NHS/EDC and PLL/GA coatings. These variations in hydrophilicity are expected to influence virus–surface interactions by modulating interfacial hydration and adsorption behavior, which may contribute to the differences in immobilization efficiency and particle morphology discussed below.^74^

Surface roughness can affect the contact angle measurement. The roughness of the bare gold (Figure S2A) and gold with SAM (Figure S2B) surface was ∼1 nm. The roughness of gold is consistent with prior studies reporting that the surface roughness of bare and functionalized gold with SAM was between 0 and 3 nm.^25^ The topographic images and height analysis of mica (Figure S2C) and mica with PLL (Figure S2D) show an average height of ∼0–1 nm. Furthermore, the control surfaces from either chemistry have a low surface roughness, between ∼0–3 nm, which is easily distinguishable from the height of the biomolecules in this study. Therefore, it was determined that surface roughness did not play a strong role in the changes in the water contact angle.

### Characterization of Tumor and Nontumor-Derived Exosomes

AFM topographic imaging was used to confirm the immobilization of tumor and nontumor-derived exosomes with NHS/EDC and PLL/GA covalent chemistries. Exosomes derived from HEK-293 (Figure 2A) and MDA-MB-231 (Figure 2B) were observed to be attached to the functionalized NHS/EDC and PLL/GA surfaces. More exosomes attached with PLL/GA were observed than by the NHS/EDC for both HEK-293 and MDA-MB-231-derived exosomes. Two-dimensional (2D) topographic images were used to generate height profiles. Representative AFM images of HEK-293-derived exosomes are shown in Figure 2C, and MDA-MB-231-derived exosomes in Figure 2D. In both cases, exosomes immobilized using PLL/GA exhibited larger height profiles than those attached with NHS/EDC, and the difference was statistically significant (*p* < 0.05, Table S2). Height distributions of the heterogeneous exosome populations were analyzed to compare the immobilization chemistries. HEK-293-derived exosomes on NHS/EDC surfaces showed smaller heights than those on PLL/GA, with PLL/GA also displaying a broader distribution of heights (Figure 2E). A similar trend was observed for MDA-MB-231-derived exosomes (Figure 2F), although the NHS/EDC surfaces had a smaller population of MDA-MB-231 exosomes. Furthermore, the size distribution of HEK-293 and MDA-MB-231-derived exosomes was left-skewed with the NHS/EDC chemistry, indicating a larger population of smaller particles. Although our main AFM studies of NHS/EDC chemistry were conducted at 4 mM SAM, a lower concentration of 2 mM SAM yielded similar results (Figure S3). In contrast, the height distributions with PLL/GA chemistry had a more Gaussian distribution for both exosome subtypes, resulting in larger sizes.

**Figure 2:**
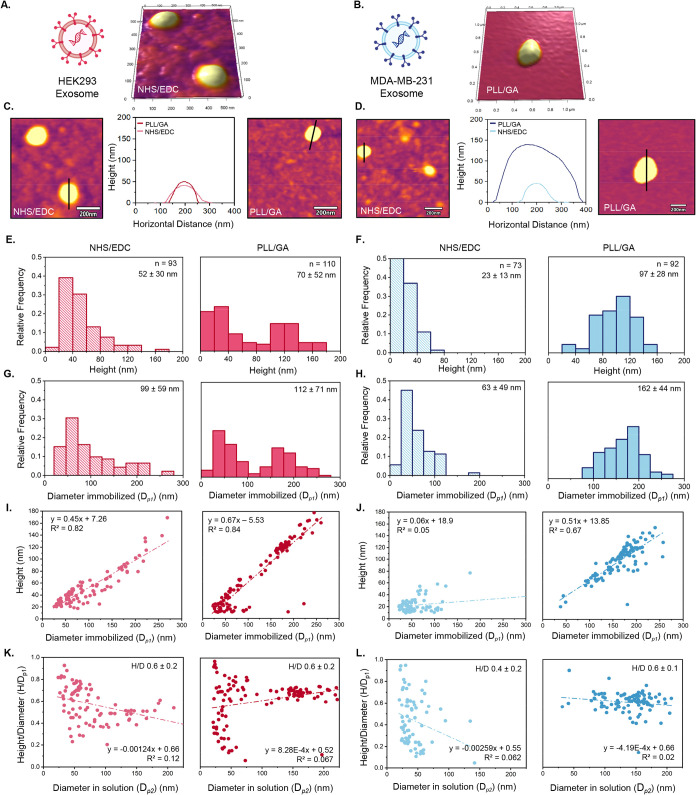
3D topographic AFM images of (A) HEK293 exosomes attached with NHS/EDC, and (B) MDA-MB-231 attached with PLL/GA at 1 × 1 μm scans under liquid conditions. (C) 2D topographic images of HEK293-derived exosomes attached with NHS/EDC (left) and PLL/GA (right) chemistry and height analysis (middle). (D) 2D topographic images of MDA-MB-231 derived exosomes attached with NHS/EDC (left) and PLL/GA (right) chemistries and height analysis (middle). (E) Histograms of HEK293-derived exosome height attached with NHS/EDC (left) and PLL/GA (right). (F) Histograms of MDA-MB-231-derived exosome height attached with NHS/EDC (left) and PLL/GA (right). (G) Histograms of HEK293-derived exosome diameters (*D_p1_*) attached with NHS/EDC (left) and PLL/GA (right). (H) Histograms of MDA-MB-231-derived exosome diameters (*D_p1_*) attached with NHS/EDC (left) and PLL/GA (right). (I) Scatter plot of the height vs diameter (*D_p1_*) of HEK293 derived exosomes attached with NHS/EDC (left) and PLL/GA (right). (J) Scatter plot of the height vs diameter (*D_p1_*) of MDA-MB-231 derived exosomes attached with NHS/EDC (left) and PLL/GA (right). (K) Scatter plot of height/diameter ratio vs diameter (*D_p2_*) in solution of HEK293 derived exosomes attached with NHS/EDC (left) and PLL/GA (right). (L) Scatter plot of height/diameter ratio vs diameter (*D_p2_*) in solution of MDA-MB-231 derived exosomes attached with NHS/EDC (left) and PLL/GA (right).

The trends for diameter were similar to those for height. We calculated the diameter of the exosomes by taking the full width at half-maximum (fwhm) height and subtracting the lateral broadening effects of the tip. Due to tip–sample convolution, AFM measurements systematically overestimate lateral particle dimensions. Lateral sizes were therefore corrected using the García model as a first-order approximation for curved nanoscale particles, including ellipsoidal virions. Particle morphology was primarily interpreted from height measurements, which are minimally influenced by probe geometry,^49,60,75^ as detailed in eqs 1–5. *D_p1_* is the diameter of the immobilized particles.^75^ The diameter was larger for the PLL/GA immobilization chemistry, and the population was skewed for the NHS/EDC chemistry to smaller particles (Figure 2 G&H). There was a statistical difference in height and overall size for MDA-MB-231–derived exosomes as a function of immobilization chemistry compared to HEK-derived exosomes (Table S2). However, in the case of HEK-derived exosomes, no significant difference in diameter was observed between the two immobilization chemistries. The diameter of exosomes is typically measured as 30 to 150 nm,^46^ and the height is typically 5 nm or larger.^76^ These dimensions can vary depending on the cell of origin and the effects of both the immobilization method and imaging conditions. HEK-293-derived exosomes immobilized to a nanoarray with PEG-lipid conjugations had a mean diameter of 25 nm and a height of 7 nm or greater under physiological conditions.^77^ In contrast, a mean diameter of 80–140 nm and heights larger than 15 nm were found for HEK-derived exosomes immobilized with antibody attachment under ambient conditions.^78^ Similar trends can be observed for MDA-MB-231-derived exosomes, in which the average diameter of exosomes with a height greater than 15 nm was 38 nm under ambient conditions and 219 nm under liquid conditions.^79^ Since this study characterized both MDA-MB-231- and HEK-293-derived exosomes by AFM under liquid conditions, the observed differences in height between the two populations are likely attributable to both the cell of origin and the type of covalent immobilization chemistry (NHS/EDC vs PLL/GA). The strong covalent amide bonds formed between substrate carboxyl groups and biomolecule surface amines likely contribute to these variations.^80^ Such conformational constraints have been previously reported to alter the structure and topography of liposomes,^81^ proteins,^82^ and enzymes^80^ due to conformational constraints imposed by the surface chemistry.^80–85^ These conformational constraints could be a result of an increase in the hydrophobicity of SAM, as shown in Figure 1D. Greater substrate hydrophobicity enhances the flattening effect of lipid bilayers by increasing the interaction of the NHS/EDC surface and the exosomes as compared to the PLL/GA surface, potentially contributing to the more pronounced structural deformation with the NHS/EDC immobilization chemistry.^74^

To assess the impact of the immobilization chemistry on exosome structural properties, we compared the height of exosomes as a function of their immobilized diameter (*D*_*p1*_). The height vs diameter of HEK-293-derived exosomes attached with NHS/EDC showed a heterogeneous distribution of exosome sizes (Figure 2I). Furthermore, the slope of the line gave insight into exosome deformation upon attachment to the functionalized surface. The slope was 0.45 for HEK-293-derived exosomes attached with NHS/EDC, indicating that exosomes with a larger height deformed more. For the PLL/GA immobilization, the slope of height versus diameter was greater, with a slope of 0.67. Thus, the PLL/GA chemistry demonstrated less deformation. For the MDA-MB-231-derived exosomes, a much lower slope of 0.06 was observed with NHS/EDC chemistry (Figure 2J); however, there was no clear trend as all exosomes appeared to shrink upon exposure to this immobilization chemistry. It is possible that the lipid membrane was disrupted during immobilization. In contrast, a higher slope of 0.51 was observed for MDA-MB-231-derived exosomes attached with PLL/GA chemistry. These findings demonstrate that the NHS/EDC chemistry has more structural effects on tumor-derived MDA-MB-231 than nontumor-derived HEK-293 exosomes. Although mechanical properties were not directly quantified, the increased deformation observed are likely due to differences in composition and mechanical properties between nontumor and tumor-derived exomes. The mechanical properties of tumor-derived exosomes are related to the malignancy level of the parent cell. Exosomes derived from malignant cell lines have a lower stiffness and are more pliable and prone to deformation than nontumor-derived exosomes.^42,49,86^ Furthermore, tumor-derived exosomes are enriched with lipids and membrane proteins,^87^ such as integrins,^88^ Alix,^89^ and tetraspanins,^90^ compared to nontumor-derived exosomes. These differences in composition allow more covalent interactions between amines on the membrane proteins and carboxylic acid groups in NHS/EDC and glutaraldehyde in PLL/GA chemistry than in nontumor-derived exosomes. Greater quantities of lipids like cholesterol, sphingolipids, and phosphatidylserine on tumor-derived exosomes lead to more hydrophobic interactions with SAM and NHS/EDC chemistry.^91–93^ It is essential to consider how differences in exosome type and composition can directly affect interactions with the immobilization chemistry and the deformation of biomolecules.

The diameters of exosomes in solution were compared to the height-to-diameter ratio of the immobilized particle to assess structural deformation resulting from covalent immobilization. The height-to-diameter ratio of the immobilized particle (*H*/*D_p1_*) was plotted vs the diameter of exosomes in solution (*D_p2_*), as calculated with eqs 6–8. The *H*/*D* ratio vs *D_p2_* plot of HEK-293-derived exosomes attached with NHS/EDC chemistry, as shown in Figure 2K, revealed a distribution of particles ranging from 25 to 250 nm in diameter. The *H*/*D* ratio averaged to be 0.6 ± 0.2 and varied considerably in smaller-sized exosomes, indicating that the flattening effect of the NHS/EDC chemistry is independent of the diameter. In contrast, larger-sized HEK-293-derived exosomes had lower *H*/*D* ratios, demonstrating more structural flattening than smaller-sized exosomes. The structural flattening of HEK293 exosomes is further supported by the negative −0.0012 slope of the *H*/*D_p1_* vs *D_p2_* line, which indicates that larger exosomes become flatter as the diameter increases. The *H*/*D* ratio vs *D_p2_* plot of HEK-293-derived exosomes attached with PLL/GA chemistry (Figure 2K) had the same *H*/*D* ratio as the NHS/EDC chemistry, indicating some flattening of the particles upon immobilization. However, a slope of zero suggests that exosomes retain more of their shape as the diameter increases. Thus, HEK-293-derived exosomes immobilized with NHS/EDC chemistry experience more flattening effects than with PLL/GA. Interestingly, the *H*/*D* ratio vs *D_p2_* plot of MDA-MB-231-derived exosomes attached with NHS/EDC (Figure 2L) showed a heterogeneous population with a smaller diameter spread and varying *H*/*D* ratios. The average *H*/*D* ratio of 0.4 ± 0.2 and a negative −0.0026 slope imply that tumor-derived exosomes are flatter upon immobilization with NHS/EDC chemistry, and these flattening effects are greater as the diameter of the exosome increases. Past research shows that tumor-derived exosomes tend to be more elastic and prone to structural deformation than healthy-derived exosomes.^94^ In contrast, the *H*/*D* ratio vs *D_p2_* plot of MDA-MB-231-derived exosomes attached with PLL/GA demonstrated a wider spread in size. Additionally, the zero slope and greater *H*/*D* ratio of 0.6 with PLL/GA indicates less structural effects than on the exosomes attached with NHS/EDC. Overall, the data suggest that both HEK-293 and MDA-MB-231-derived exosomes are affected less by the PLL/GA immobilization chemistry than the NHS/EDC chemistry, which may either selectively immobilize smaller biomolecules or induce deformation or fragmentation in larger, softer particles.

### Characterization of Enveloped Viruses

AFM topographic imaging confirmed the successful covalent immobilization of both enveloped viruses—SuHV and XMuLV—using NHS/EDC and PLL/GA surface chemistries. Distinct differences in particle morphology and immobilization density were observed between the two methods. Height profiling was conducted on 2D topographical images acquired under physiological conditions for SuHV (Figure 3A,C) and XMuLV (Figure 3B,D). Similar to trends observed for exosomes, both viruses appeared smaller when immobilized via NHS/EDC than PLL/GA (Figure 3E,F). The average particle heights obtained for SuHV and XMuLV on PLL/GA surfaces were consistent with their expected native dimensions, around 100–200 nm for SuHV^67^ and 80–120 nm for XMuLV.^66^ After correcting for AFM tip broadening effects (eqs 1–5), the calculated particle diameters (*D_p1_*) further supported these observations. For both SuHV (Figure 3G) and XMuLV (Figure 3H), the apparent diameters were greater on PLL/GA-functionalized surfaces than on NHS/EDC. Moreover, NHS/EDC immobilization produced left-skewed diameter distributions for both viruses, whereas PLL/GA yielded distributions approaching a normal Gaussian profile, consistent with a more uniform immobilization. Quantitative analysis also revealed substantial differences in immobilization efficiency between the two chemistries. For SuHV, only 14 particles were detected across six 5 × 5 μm AFM scan areas on NHS/EDC, compared to 106 particles on PLL/GA. XMuLV exhibited a similar pattern, with 32 particles detected on NHS/EDC versus 106 on PLL/GA. Even with the low number of immobilized particles for NHS/EDC for SuHV and XMuLV, there was a statistically significant difference between height and diameter profile for both SuHV and XMuLV immobilized on NHS/EDC and PLL/GA chemistries, as shown in Table S2. The differences in morphology and immobilization efficiency observed between NHS/EDC and PLL/GA surfaces emphasize the importance of immobilization mechanism in governing virus–surface interactions. Although both chemistries produce covalent attachment, NHS/EDC promotes direct amide bond formation with envelope-associated proteins, creating localized anchoring points that restrict membrane mobility and promote partial flattening of the lipid envelope.^80,84^ This localized binding likely contributes to the reduced particle heights and left-skewed size distributions observed for both SuHV and XMuLV, indicating heterogeneous deformation during immobilization.^82^ The NHS/EDC chemistry also has a lower efficiency of immobilization for the enveloped viruses. Consistent with observations for model exosomes in this study, increased SAM hydrophobicity may further promote lipid flattening, contributing to the observed structural changes.^78,80–85^ Collectively, these results demonstrate that PLL/GA chemistry provides significantly higher immobilization density and more native-like viral morphology for both enveloped viruses.

**Figure 3:**
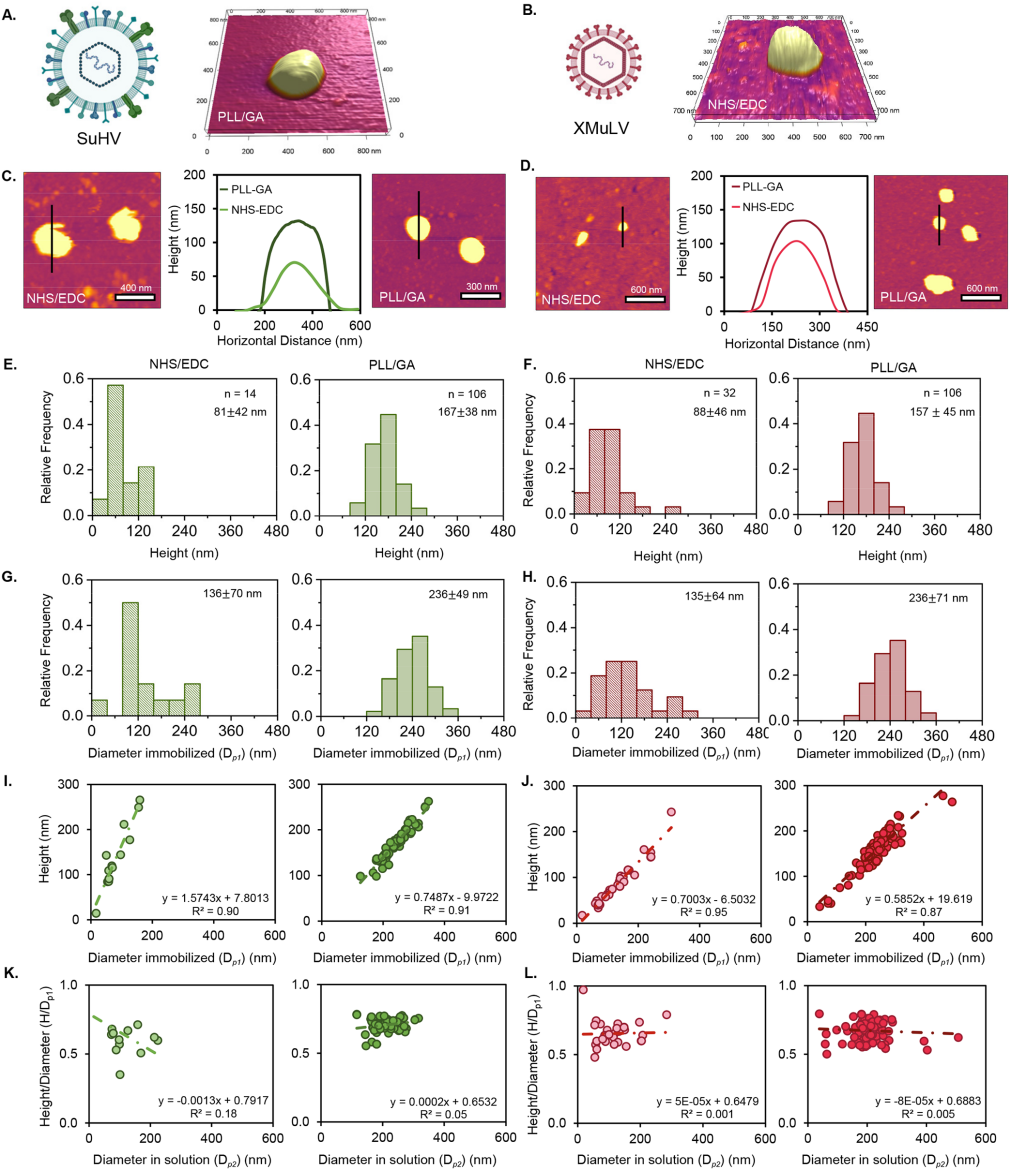
3D topographic AFM images of (A) SuHV immobilized on PLL/GA and (B) XMuLV on NHS/EDC at 1 × 1 μm scans under liquid conditions. (C) 2D topographic images of SuHV attached with NHS/EDC (left) and PLL/GA (right) chemistry and height analysis (middle). (D) 2D topographic images of XMuLV attached with NHS/EDC (left) and PLL/GA (right) chemistries and height analysis (middle). (E) Histograms of SuHV height attached with NHS/EDC (left) and PLL/GA (right). (F) Histograms of XMuLV height attached with NHS/EDC (left) and PLL/GA (right). (G) Histograms of SuHV diameter (*D_p1_*) attached with NHS/EDC (left) and PLL/GA (right). (H) Histograms of XMuLV diameter (*D_p1_*) attached with NHS/EDC (left) and PLL/GA (right). (I) Scatter plot of the height vs diameter (*D_p1_*) of SuHV attached with NHS/EDC (left) and PLL/GA (right). (J) Scatter plot of the height vs diameter (*D_p1_*) of XMuLV attached with NHS/EDC (left) and PLL/GA (right). (K) Scatter plot of height/diameter ratio vs diameter (*D_p2_*) in solution of SuHV attached with NHS/EDC (left) and PLL/GA (right). (L) Scatter plot of height/diameter ratio vs diameter (*D_p2_*) in solution of XMuLV attached with NHS/EDC (left) and PLL/GA (right).

To further assess covalent immobilization effects on SuHV and XMuLV, viral morphology was quantitatively analyzed. A positive correlation was observed between height and the immobilized diameter (*D_p1_*) plots for both NHS/EDC and PLL/GA chemistries, indicating that taller particles generally exhibited larger diameters (Figure 3I&J). For SuHV immobilized on NHS/EDC (Figure 3I), the slope for height vs *D_p1_* is 1.5, suggesting that these particles largely retained their native morphology. However, the average particle height was around 81 ± 42 nm, which is less than the average diameter of a fully intact virus, which is around 150–200 nm.^67^ It is possible that the envelope was removed from the SuHV during immobilization and only the capsid was immobilized. This implies that covalent immobilization with NHS/EDC disrupts the lipid bilayer while preserving the protein capsid, consistent with the capsid dimensions reported for SuHV.^95^ In contrast, SuHV immobilized on PLL/GA exhibited heights closer to those of intact particles (167 ± 38 nm), with a slope of height vs *D_p1_* of 0.75, indicating that PLL/GA immobilization better preserves the native morphology of SuHV with minimal structural deformation.^95^ Although the slope for XMuLV immobilization was slightly higher on NHS/EDC (0.7) compared to PLL/GA (0.6), showing that the effect of immobilization on deformation for XMuLV is similar for each chemistry. To further evaluate the extent of particle flattening upon immobilization, the *H*/*D_p1_* ratio of immobilized particles was plotted against the diameter of viruses in solution (*D_p2_*). An *H*/*D*_*p1*_ ratio close to 1 indicates a more spherical geometry upon attachment, while lower values reflect greater flattening or deformation of the particle on the surface. For both viruses, particles immobilized with NHS/EDC showed consistently lower height-to-diameter (*H*/*D*) ratios compared to PLL/GA (Figure 3K&L), indicating more pronounced deformation. Quantitatively, SuHV immobilized with NHS/EDC had an average *H*/*D* ratio of 0.64 ± 0.18, while PLL/GA immobilization preserved a more spherical shape with an *H*/*D* ratio of 0.70 ± 0.04 (Figure 3F). Similarly, XMuLV displayed slightly lower *H*/*D* ratios for NHS/EDC (0.65 ± 0.01) compared to PLL/GA (0.67 ± 0.07), though the difference was less pronounced than with SuHV. There was no change in *H*/*D* ratio as a function of virus diameter in liquid for any virus or immobilization chemistry. Among the two enveloped viruses, differences in deformation responses between XMuLV and SuHV suggest that variations in particle mechanical properties influence sensitivity to immobilization chemistry. The increased sensitivity of SuHV to deformation compared to XMuLV is likely attributed to structural differences between the two viruses, particularly the presence of a tegument layer in SuHV, as shown in Figure 1B. This amorphous, protein-rich layer, similar to that found in herpesviruses like HSV-1, acts as a flexible buffer between the capsid and the envelope, aiding viral assembly and host interactions.^96^ However, its soft, gel-like nature makes it mechanically sensitive,^97^ prone to collapse or spreading under external forces such as those applied during immobilization or AFM scanning. In contrast, XMuLV lacks this intermediate layer and instead features a more compact core and matrix that confer greater mechanical stability.^98^ These observations underscore how virus-specific architectures influence morphological integrity during surface-based imaging.

### Characterization of a Nonenveloped Viruses

PPV, a model nonenveloped virus, was effectively immobilized using both NHS/EDC and PLL/GA chemistries. 2D topographical AFM image of PPV particles bound to these functionalized surfaces (Figure 4A,B) showed comparable particle coverage, with approximately 100 particles detected across six scanned frames. Quantitative AFM analysis showed that PPV particles exhibited comparable height profiles following immobilization on both NHS/EDC- and PLL/GA-functionalized surfaces (Figure 4C). In contrast, differences were observed in the diameter (Figure 4D) of particles immobilized on NHS/EDC, which exhibited larger apparent diameters as compared to those on PLL/GA. Similar height profiles suggest preserved particle size, while the larger apparent diameters on NHS/EDC surfaces likely arise from particle aggregation, causing neighboring virions to appear as single enlarged features in AFM images.^99^ We did not image at the resolution that would allow individual, aggregated particles to be observed. Both chemistries provided robust and consistent particle attachment, with similar height measurements across replicates. This observation contrasts with the behavior of the enveloped viruses examined, which displayed chemistry-dependent deformation, and aligns with previous studies reporting comparable physicochemical characteristics of PPV under similar AFM immobilization conditions.^25,100^ However, notable differences in diameter (*D_p1_*) were observed. Particles immobilized with NHS/EDC appeared wider in diameter (47 ± 24 nm) than those on PLL/GA (27 ± 18 nm), suggesting possible partial aggregation during covalent attachment, as shown in the 2D topographical image in Figure 4B. The increased apparent diameters resulted in lower *H* vs *D_p1_* slopes for both NHS/EDC (0.31) and PLL/GA (0.38). For both immobilization chemistries, the slopes near 0.3 indicate that the particles experienced measurable deformation upon surface immobilization, consistent with lateral compression or height reduction associated with adsorption and tip–sample interactions. Further structural deformation was characterized with the *H*/*D_p1_* ratio vs *D_p2_*. As shown in Figure 4F, the NHS/EDC-immobilized PPV particles exhibited a lower average *H*/*D* ratio of 0.59 ± 0.20; in contrast, the average *H*/*D* ratio for PPV immobilized on PLL/GA was 0.99 ± 0.41, indicating a near-spherical geometry upon attachment. Considering that the expected PPV dimensions range from 20–30 nm in both height and diameter^68^ the increased apparent diameter and reduced *H*/*D* ratio observed for NHS/EDC suggest enhanced particle deformation or potential aggregation as can be seen in Figure 4B,D. Our findings indicate that PLL/GA chemistry affords more uniform and representative immobilization of PPV particles, better preserving their native morphology for high-resolution topographical analyses.

**Figure 4:**
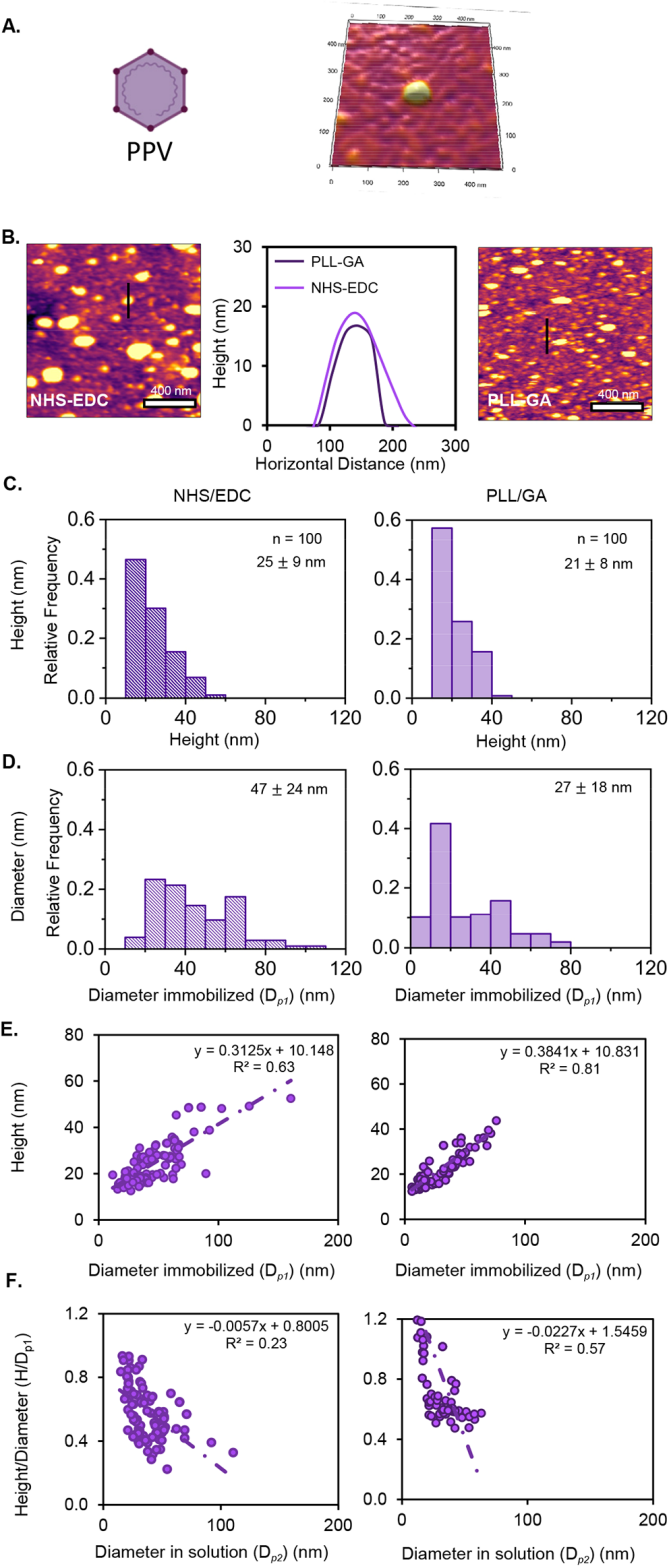
3D topographic AFM images of (A) PPV immobilized on NHS/EDC and PLL/GA at 1 × 1 μm scans under liquid conditions. (B) 2D topographic image of PPV attached with NHS/EDC (left) and PLL/GA (right) chemistry and height analysis (middle). (C) Histograms of PPV height immobilized with NHS/EDC (left) and PLL/GA (right). (D) Histograms of PPV diameter (*D_p1_*) immobilized with NHS/EDC (left) and PLL/GA (right). (E) Scatter plot of the height vs diameter (*D_p1_*) of PPV immobilized with NHS/EDC (left), and PLL/GA (right). (F) Scatter plot of height/diameter ratio vs diameter (*D_p2_*) in solution of PPV attached with NHS/EDC (left) and PLL/GA (right).

### Secondary Characterization of Biomolecules

The morphology of the particles in liquid was characterized to compare the biomolecule population prior to immobilization. Liquid phase TEM indicated that the HEK-293 and MDA-MB-231-derived exosomes ranged in size from 60–200 nm (Figure 5A). The model viruses exhibited morphology and size consistent with their expected characteristics. PPV, the nonenveloped model virus, displayed uniform spherical particles with diameters ranging between 26–32 nm.^68^

**Figure 5:**
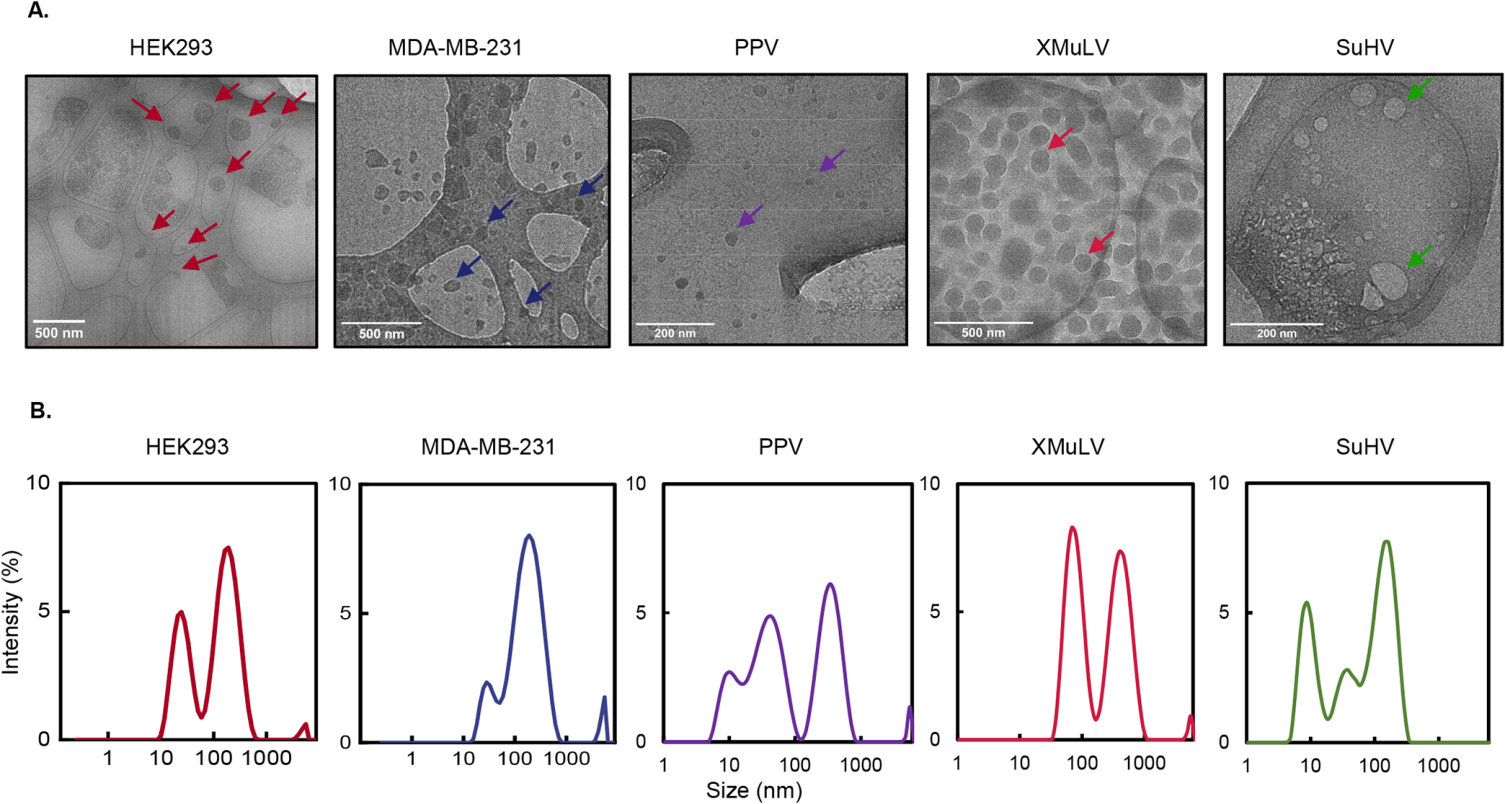
Transmission electron microscopy (TEM) and dynamic light scattering (DLS) of biomolecules. (A) Liquid cell TEM images of exosomes HEK293 and MDA-MB-231 derived exosomes of various sizes and viruses PPV, XMuLV, and SuHV. (B) Particle size analysis with DLS of HEK293 and MDA-MB-231 exosomes and viruses PPV, XMuLV, and SuHV.

The particle size distributions were also analyzed with DLS. The size distributions of MDA-MB-231 and HEK-293-derived exosomes were determined from the primary DLS peak to be 216.93 ± 19.13 and 190.87 ± 22.38 nm, respectively (Figure 5B), in agreement with the TEM results. Overall, the characterization shows variability in the exosome size, which is common due to the heterogeneity of exosomes and the influence of the applied isolation and characterization methods.^101^ For example, smaller-sized HEK-293-derived exosomes were isolated by precipitation, with diameters of 60 nm for DLS and 50–80 nm for SEM and TEM.^102^ In contrast, HEK-293-derived exosomes isolated by ultracentrifugation were 140–158 nm in diameter.^103^ Furthermore, the heterogeneous size range of exosomes can be challenging to measure with DLS due to the high polydispersity index of the sample, skewing toward larger-sized vesicles.^104^

The size distribution of the viruses varied by virus type. The enveloped virus SuHV exhibited a broader size distribution, with an average diameter of 147 ± 50 nm, and for XMuLV, the peak was 83 ± 17 nm (Figure 5B), consistent with previous reports.^66,67^ In the case of nonenveloped PPV, the primary peak was observed at 45 ± 20 nm, aligning with the literature.^68^

Overall, the characterization of exosomes and viral size and morphology was performed using TEM, DLS, and AFM under liquid conditions. The slight differences in average exosome diameter between these methods are attributable to methodological limitations. A slightly larger but overlapping diameter size was observed in DLS for both HEK-293 and MDA-MB-231 exosomes compared to TEM and AFM, due to a highly polydisperse sample. Additionally, the observed TEM average diameter of 60–200 nm for exosomes overlapped with the AFM diameter calculated for the exosome in suspension (*D_p2_*), with averages of 40–80 nm for NHS/EDC and 90–140 nm for PLL/GA. Imaging artifacts and the AFM immobilization method can slightly alter exosome size. The overall morphological and size analysis validated the structural integrity of the samples and supported our AFM-based morphological analysis.

## Conclusion

Exosomes and viruses were covalently immobilized to gold coated surfaces with a SAM using NHS-EDC chemistry, and mica functionalized with PLL using GA as a cross-linker. By comparing NHS/EDC chemistry on SAM-functionalized gold and PLL/GA on mica, we demonstrate that PLL/GA provides a more robust platform for immobilizing a wide range of biomolecules, including exosomes and viruses, preserving their native morphology and ensuring higher particle retention. In contrast, NHS/EDC chemistry selectively immobilizes smaller biomolecules and induces deformation or fragmentation in larger, softer particles, as evidenced by reduced height-to-diameter ratios and lower particle densities.

Our results further demonstrate that the structural properties of biomolecules strongly influence their response to surface chemistry. Lipid-enveloped systems, including exosomes and enveloped viruses, were more prone to deformation upon immobilization, whereas the nonenveloped virus, characterized by a rigid protein capsid, largely preserved its structural integrity across both chemistries. Notably, tumor-derived MDA-MB-231 exosomes exhibited greater flattening and structural disruption compared to HEK-293 exosomes, underscoring the combined influence of particle size, mechanical compliance, and immobilization strategy. Consistent with these observations, AFM analysis revealed that immobilization chemistry significantly affects viral morphology, with increased ellipsoidal deformation indicating partial flattening upon adsorption. Convolution-corrected height-to-diameter analysis confirmed that these changes arise primarily from surface-induced deformation rather than imaging artifacts, highlighting the critical role of the immobilization strategy in preserving native nanoscale structure.

Overall, this work emphasizes that the choice of covalent chemistry is not only a technical consideration but also a critical factor that can influence the accuracy of single-particle characterization studies. By providing insights into how immobilization strategies affect particle morphology and retention, our study offers a framework for selecting the most suitable surface chemistry based on particle type, size, and mechanical properties. These findings advance the understanding of nanoscale biological particle interactions with surfaces and provide guidance for AFM-based studies in virology, extracellular vesicle research, and nanobiotechnology applications, ensuring that structural and mechanical analyses reflect the true properties of the biomolecules under investigation.

## Supplementary Material



## Notes

AFM-based height analysis models, control surface topography, roughness characterization, contact angle measurements of functionalized surfaces, and statistical analysis of biomolecule height and diameter (PDF)
