# Supplementary material for: Physical Changes of Biomacromolecules upon Covalent Surface Immobilization
Source: Langmuir. 2026 Mar 12;42(11):8018–31. doi: 10.1021/acs.langmuir.5c06836 (PMC13019682; doi:10.1021/acs.langmuir.5c06836)
Supplement: Supplementary file 1 [file la5c06836_si_001.pdf]

Supplementary Information:

Physical Changes of Biomacromolecules Upon Covalent Surface Immobilization

Bianca Mercado Velez[1,2#], Vaishali Sharma[1,2#], Seth Kriz[2,3], Erico T. F. Freitas[4], Paul Goetsch[1,2], Caryn L. Heldt[2,3*]

[1]Department of Biological Sciences, Michigan Technological University, Houghton, MI

[2]Health Research Institute, Michigan Technological University, Houghton, MI

[3]Department of Chemical Engineering, Michigan Technological University, Houghton, MI

[4]Materials Characterization and Fabrication Facility, Michigan Technological University, Houghton, MI

[#] Authors have contributed equally

*Corresponding author: 1400 Townsend Dr., Houghton, MI 49931, USA

heldt@mtu.edu, phone (906)487-1134, fax (906)487-3213

Raw data found at: https://digitalcommons.mtu.edu/all-datasets/69

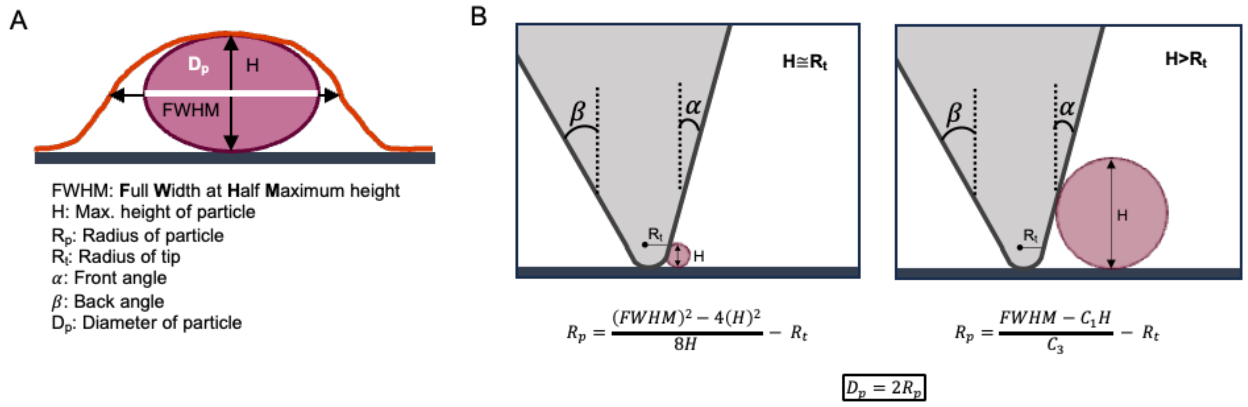

Figure S1. Equations used to determine the population height and diameter of attached biomolecules using AFM-generated height profiles. (A) Schematic of FWHM determination of an attached biomolecule based on the max height and angles of the AFM tip. (B) Garcia model, when ($H>R_t$) and when ($H \cong R_t$).

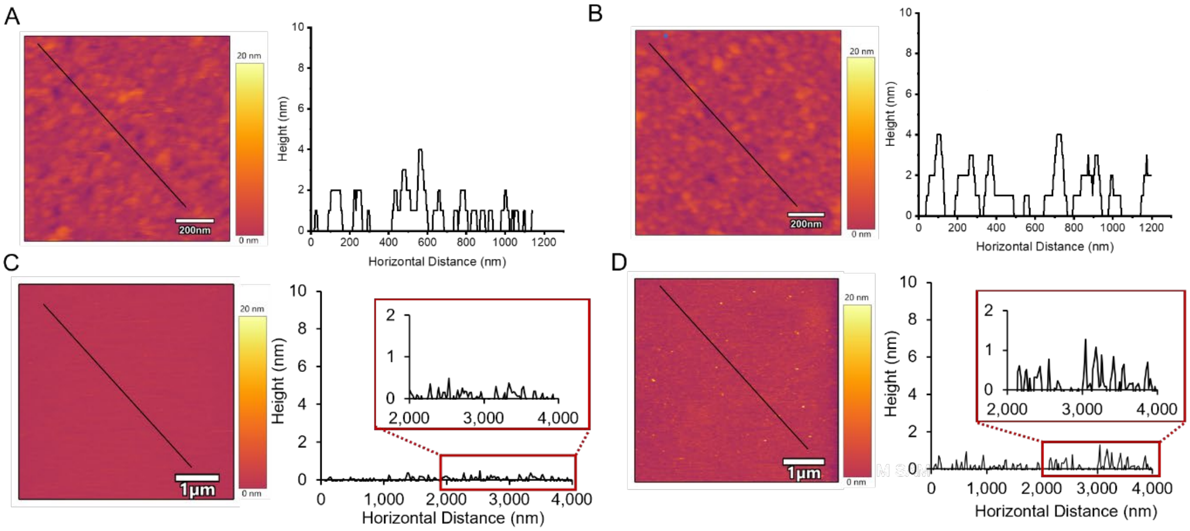

Figure S2. Topographic images and height analysis of control surfaces. NHS/EDC control surfaces (A) bare gold and (B) gold with SAM are 1x1 µm scans. PLL/GA control surfaces (C) mica and (D) mica with PLL are 5x5 µm scans.

Table S1. Average root mean square (RMS) roughness values of control surfaces: bare gold, gold with SAM at 1x1 μm scans, and freshly cleaved mica and mica with PLL.

**Surface:**	**RMS Roughness (nm)**	**Measurement Method**	**Scan Area (um x um)**
Bare Gold	1.40 ± 0.12	AFM	1 x 1
Gold/SAM	1.53 ± 0.30	AFM	1 x 1
Freshly cleaved Mica	0.18 ± 0.04	AFM	1 x 1
Mica/PLL	0.76 ± 0.25	AFM	1 x 1

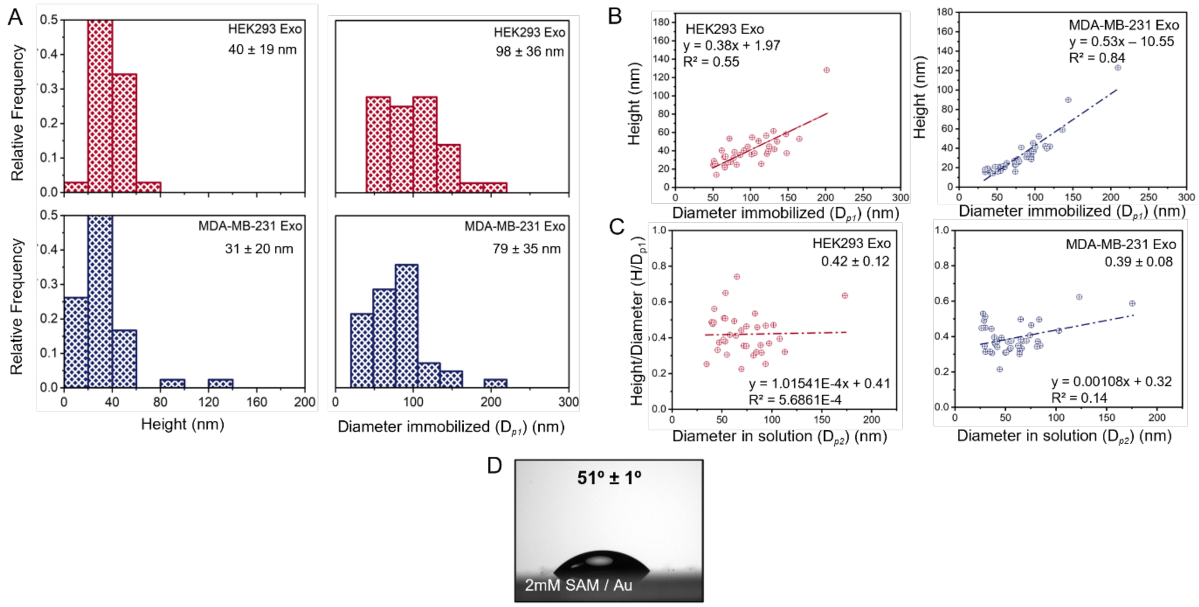

Figure S3. (A) Height and diameter histograms of HEK293 and MDA-MB-231 derived exosomes immobilized with gold functionalized with 2mM SAM and NHS/EDC. (B) A scatter plot of the height vs. diameter of exosomes HEK293 and MDA-MB-231 immobilized with Gold functionalized with SAM and NHS/EDC, and mica functionalized with PPL and GA was plotted for deformation correlation (C). Scatter plots of height/diameter ratio vs. diameter of exosomes HEK293 and MDA-MB-231 immobilized with Gold functionalized with 2 mM SAM and NHS/EDC. (D) Contact angle measurement of water on the control surface, gold functionalized with a 2 mM SAM containing NHS/EDC.

Table S2: Statistical analysis of biomolecule height and diameter on covalently functionalized surfaces

Biomolecules	Statistical analysis conditions	p-value
HEK293	Height on NHS/EDC vs PLL/GA	3.1E-3
	Diameter on NHS/EDC vs PLL/GA	**0.15**
MDA-MB-231	Height on NHS/EDC vs PLL/GA	2.5E-48
	Diameter on NHS/EDC vs PLL/GA	3.6E-27
SuHV	Height on NHS/EDC vs PLL/GA	2.0E-6
	Diameter on NHS/EDC vs PLL/GA	1.0E-4
XMuLV	Height on NHS/EDC vs PLL/GA	1.0E-9
	Diameter on NHS/EDC vs PLL/GA	4.0E-10
PPV	Height on NHS/EDC vs PLL/GA	3.7E-3
	Diameter on NHS/EDC vs PLL/GA	2.0E-10
